# Extracting Phonetic Features From Natural Classes: A Mismatch Negativity Study of Mandarin Chinese Retroflex Consonants

**DOI:** 10.3389/fnhum.2021.609898

**Published:** 2021-03-24

**Authors:** Zhanao Fu, Philip J. Monahan

**Affiliations:** ^1^Department of Linguistics, University of Toronto, Toronto, ON, Canada; ^2^Department of Language Studies, University of Toronto Scarborough, Toronto, ON, Canada; ^3^Department of Psychology, University of Toronto Scarborough, Toronto, ON, Canada

**Keywords:** mismatch negativity (MMN), retroflex, Chinese, EEG – electroencephalogram, speech perception, phonology, phonetics, phonetic features

## Abstract

How speech sounds are represented in the brain is not fully understood. The mismatch negativity (MMN) has proven to be a powerful tool in this regard. The MMN event-related potential is elicited by a deviant stimulus embedded within a series of repeating standard stimuli. Listeners construct auditory memory representations of these standards despite acoustic variability. In most designs that test speech sounds, however, this variation is typically intra-category: All standards belong to the same phonetic category. In the current paper, inter-category variation is presented in the standards. These standards vary in manner of articulation but share a common phonetic feature. In the standard retroflex experimental block, Mandarin Chinese speaking participants are presented with a series of “standard” consonants that share the feature [retroflex], interrupted by infrequent non-retroflex deviants. In the non-retroflex standard experimental block, non-retroflex standards are interrupted by infrequent retroflex deviants. The within-block MMN was calculated, as was the identity MMN (iMMN) to account for intrinsic differences in responses to the stimuli. We only observed a within-block MMN to the non-retroflex deviant embedded in the standard retroflex block. This suggests that listeners extract [retroflex] despite significant inter-category variation. In the non-retroflex standard block, because there is little on which to base a coherent auditory memory representation, no within-block MMN was observed. The iMMN to the retroflex was observed in a late time-window at centro-parieto-occipital electrode sites instead of fronto-central electrodes, where the MMN is typically observed, potentially reflecting the increased difficulty posed by the added variation in the standards. In short, participants can construct auditory memory representations despite significant acoustic and inter-category phonological variation so long as a shared phonetic feature binds them together.

## Introduction

Speech is a variable and continuous signal. Despite this, successful spoken word recognition requires listeners to identify and extract meaningful linguistic units. Different models rely on different linguistic units, from syllables (Greenberg, 1999) to phonological features (Stevens, 2002) to a combination of both (Hickok, 2014). In particular, features play a central role in several models of speech processing (Halle and Stevens, 1962; McClelland and Elman, 1986; Stevens, 2002; Gow, 2003; Poeppel and Monahan, 2011). Stevens (2002) proposed that listeners utilize features to identify major landmarks in the speech signal. The identification of these features is critical for word segmentation and lexical access. Despite their central role in speech processing models and phonological theory (Clements and Hume, 1995; Halle, 2002), evidence that the perceptual system or human brain utilizes features or feature-like representations has been difficult to establish. Their best support has arisen from neurophysiology (see Monahan, 2018 for a review).

Individual speech sounds are a complex constellation of articulatory and acoustic properties. Phonological theory has long represented these properties with distinctive features (Jakobson et al., 1961; Chomsky and Halle, 1968; Trubetskoy, 1969; Clements, 1985; McCarthy, 1988). Features encode the relation between an aspect of a speech sound’s articulation and the corresponding acoustic signature (Halle, 1983; Baković, 2014). Moreover, they also serve to denote active natural classes, that is, sets of sounds that pattern together in the phonological grammar. Initially, features were binary in nature, and each feature had a polarity: A consonant was either [+obstruent] or [−obstruent] (Chomsky and Halle, 1968). These binary feature systems, however, wrongly predicted that both positive and negative specifications should denote active natural classes in the grammar (van der Hulst, 2016). For example, some languages have word-final devoicing of voiced obstruents (e.g., German, Dutch), while others allow both voiced and voiceless obstruents in word-final position (e.g., English). No language, as far as we know, employs a word-final voicing rule (Reiss, 2017; although see Blevins et al., 2020 for a potential recent counterexample).

As such, privative, or monovalent, features were proposed (Clements, 1985; Sagey, 1986; Harris and Lindsey, 1995; Lombardi, 1995). In a privative system, a nasal segment contains the feature [nasal], whereas a non-nasal segment completely lacks a representation for nasality in memory. Underspecification accounts go one step further and posit that only predictable privative features are stored (Mester and Itô, 1989; Steriade, 1995). As an example, the place of articulation for the coronal nasal segment [n] is often determined by its local phonotactic context, as it assimilates in place to match the following consonant; otherwise, it has the putative default place of articulation [coronal]. Given this predictability, the feature [coronal] is argued to be underspecified in memory (Archangeli, 1988; Avery and Rice, 1989).

Neurophysiological measures [e.g., electroencephalography (EEG), electrocorticography (ECoG), magnetoencephalography (MEG)] have been used to assess the nature of speech sound representations (Näätänen, 2001; Mesgarani et al., 2014). An extensively used method is the mismatch negativity (MMN, mismatch field (MMF/MMNm) in MEG; Näätänen et al., 2007; Näätänen and Kreegipuu, 2012). The MMN is a negative deflection in the event-related potential (ERP) to an infrequent deviant stimulus embedded within a sequence of repeating standard stimuli. It peaks between 100 ms and 400 ms post-stimulus onset and in EEG, is largest over fronto-central electrode sites. Auditory cortex is the cortical source of the auditory MMN, and its precise location depends on the property of the deviant that differs from that of the standards (e.g., frequency, intensity, duration; see Alho, 1995 for a review). For speech stimuli, the MMN localizes to supratemporal auditory cortex (Aulanko et al., 1993). In studies of speech perception, the “varying standards” paradigm is often utilized. There, different acoustic tokens of the standard are used that all belong to the same speech sound category. This encourages participants to construct auditory memory representations of the standards that are not based solely on acoustic properties but instead reflect phonetic or phonological categories (Phillips et al., 2000; Kazanina et al., 2006; Hestvik et al., 2020).

A listener’s native language phonology modulates the size and/or presence of the MMN (Näätänen et al., 1997; Winkler et al., 1999, 2003; Sharma and Dorman, 2000; Kazanina et al., 2006; Nenonen et al., 2003; Ylinen et al., 2006; K. Yu et al., 2019). Additionally, various MMN results support a role for phonological features during speech processing (Eulitz and Lahiri, 2004; Scharinger et al., 2012; Cornell et al., 2013; Hestvik and Durvasula, 2016; Scharinger et al., 2016b; Schluter et al., 2016). In a number of these studies, an asymmetric MMN is observed. In most MMN designs, two categories are tested, and participants are presented with two experimental blocks separated by a short break in a single testing session. In the first experimental block, one category is the standard while the other is the deviant. This role is reversed in the second experimental block. The asymmetry is that one deviant elicits a larger MMN than the other deviant. These asymmetries are often taken to reflect the underlying featural content of the two categories consistent with underspecified representations (Lahiri and Reetz, 2002, 2010). A larger MMN is observed when the standard is specified for a given feature and the deviant mismatches with that feature. When the standard is underspecified, there is no mismatch between the standard and deviant, and as such, a smaller or no MMN is observed. Asymmetric MMN results have been observed for vowels (Eulitz and Lahiri, 2004; Cornell et al., 2011; Scharinger et al., 2012, 2016b), consonants (Cornell et al., 2013; Hestvik and Durvasula, 2016; Schluter et al., 2016; Hestvik et al., 2020) and lexical tones (Politzer-Ahles et al., 2016).

In all these studies, however, a single category is used for the standards. In the current paper, we present multiple different phonetic categories in the standards that all share the common phonetic feature [retroflex]. Gomes et al. (1995) reported that when sinusoidal standards shared the same duration but varied in intensity and frequency, an MMN was still observed to deviants that differed along all three parameters. These findings suggest that listeners can extract single cues, in this case duration, from the standards and build an auditory memory representation based on a single cue. Most phonetic and phonological features refer to a single cue that denotes natural classes of sounds (Halle, 2002), while some features refer to multiple acoustic cues that denote a single natural class (e.g., retroflex; Hussain et al., 2017). The question this paper addresses is whether listeners also extract features from standards that belong to the same natural class and use those features to construct auditory memory representations.

Standard Mandarin Chinese has a relatively rich set of retroflex consonants at the coronal place of articulation. In particular, Mandarin Chinese has the fricative [ʂ], affricate [tʂ], aspirated affricate [tʂ^h^], and a final category [ʐ∼ɻ]. This last category has been argued to be a voiced fricative (Duanmu, 2007), while others have argued that phonetically, it is an approximant (Lee and Zee, 2003; Lee-Kim, 2014; Lin, 2007). Mandarin Chinese also has the non-retroflex coronal counterparts for each of these categories: [s], [ts], [ts^h^] and [l], respectively. Here, each of these sound categories is presented both as standards and deviants, which makes the current study a unique departure from traditional MMN studies, where intra-category stimulus tokens are used.

The current paper describes the results of a single MMN experiment using EEG with Mandarin Chinese retroflex consonants. Most previous MMN studies of features assume a privative feature system and argue for underspecified featural representations (see above). Here, we spell out the predictions for both binary and privative accounts assuming listeners can extract constant phonetic properties despite inter-category variation in the standards. Overall, we attempt to determine whether the property (or lack thereof) retroflex, which gives speech sounds an “r”-color, is extracted by Mandarin listeners and used to construct an auditory memory representation. In the retroflex standard experimental block, listeners heard the standards [ʂ tʂ tʂ^h^ ɻ] interrupted by an occasional deviant stimulus, e.g., [s ts ts^h^ l]. In the non-retroflex standard experimental block, the standard-deviant relationship was reversed. All segments used in the experiment are [coronal] and as such, the feature [coronal] is insufficient to explain the presence of an MMN. In a binary feature account, listeners should extract [+retroflex] in the retroflex standard block and [−retroflex] in the non-retroflex standard block. Equal-sized MMNs are predicted for both blocks as positive and negative feature valences are equally informative. Under a privative account, however, only retroflex consonants are stored with the feature [retroflex] in their auditory memory representation. Then, we predict an asymmetric MMN. We anticipate an MMN only in the retroflex standard block as listeners extract the feature [retroflex]. Because the standards in the non-retroflex standard block do not share a common feature to the exclusion of the deviants under a privative account, we do not anticipate observing a clear MMN.

## Methods

### Participants

Thirty-three right-handed native Mandarin speakers participated in the experiment. All subjects were recruited from the University of Toronto Scarborough. The entire experimental session was conducted in Mandarin Chinese. No participant reported any hearing, language, or neurological deficits. Data from seven participants were excluded due to technical issues during the recording sessions. This left 26 participants (17 females, mean (*x̄*) age = 19.7 years, standard deviation (*s*) = 0.7 years, *x̄* age of arrival = 16.8 years, *s* = 2.3 years). All participants also spoke English. Nine participants reported proficiency in additional languages (i.e., Cantonese, Shanghainese, and Japanese). All participants self-reported 10/10 on listening proficiency and at least 8/10 in speaking proficiency in Mandarin, except for one participant, who self-rated 7/10 in listening proficiency. The Mandarin participants reported consistently lower speaking and listening English self-ratings (speaking: *x̄* = 6.54, *s* = 1.64; listening: *x̄* = 7.43, *s* = 1.56) and only reported using English on average 23.7% (*s* = 18%) of the time in their daily lives. The mean length of stay in Canada was 3.02 years (*s* = 2.63 years). The experiment was approved by the University of Toronto Research Ethics Board. All participants provided written informed consent and received course credit.

### Stimuli

Stimuli included eight [Cɤː4] syllables. The C represents a consonant from four retroflex/non-retroflex consonant pairs: [s]/[ʂ], [ts]/[tʂ], [ts^h^]/[tʂ^h^], [l]/[ɻ]. The eight consonants represent every retroflex consonant in the Mandarin inventory and their non-retroflex counterparts. The high-mid back unrounded vowel [ɤ] and Tone 4 were chosen to form the frame because they yield phonotactically legal syllables with all consonants in the experiment, and all were real words of Mandarin Chinese. For example, [sɤː4] corresponds to the words 色 “color” or 涩 “astringent”. Some [Cɤː4] stimuli corresponded to multiple lexical items. The decision was taken to use real words. The relatively limited Mandarin syllable inventory made it impossible to obtain a set of CV syllables where each was a phonotactically legal pseudoword in the language across all eight consonant, vowel and tone combinations. The alternatives were to use a set of stimuli that contained a mixture of words and pseudowords or a set of eight phonotactically illegal syllables. Choosing a mixture of words and pseudowords would potentially result in evoking a set of processes (e.g., lexical access) that would be present for some items but not others. Meanwhile, using phonotactically illegal syllables would require participants to employ repair strategies that are not part of natural language processing in their native language (Dehaene-Lambertz et al., 2000).

Stimuli were produced by a male native speaker of Mandarin Chinese. The tokens were recorded with an Audio-Technica AT3035 cardioid microphone onto a MixPre-3 digital recorder (Sound Devices LLC, United States). The stimulus recording session occurred in a sound-attenuated booth. The audio files were recorded with a 44.1 kHz sampling frequency at 16-bit depth. The retroflex stimuli had a mean duration of 429 ms (*s* = 9 ms). The non-retroflex stimuli had a mean duration of 459 ms (*s* = 81 ms). Table 1 provides the syllable and consonant durations for the stimuli in our experiment, the number of words and senses for each syllable, and their corpus frequencies. The number of words and senses are obtained from Xinhua dictionary (The Commercial Press, 2009). Word frequencies are obtained from DoWLS (Neergaard et al., 2016), which is based on SUBTLEX-CH (Cai and Brysbaert, 2010) and includes phonetic transcriptions. Cosine^2^ offset ramps were applied to the final 10 ms of each stimulus. Stimulus intensity was normalized to 70 dB SPL.

**TABLE 1:**
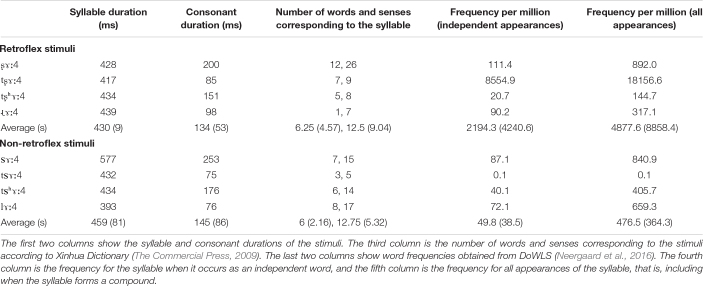
Acoustic properties and lexical information of the stimuli.

**FIGURE 1:**
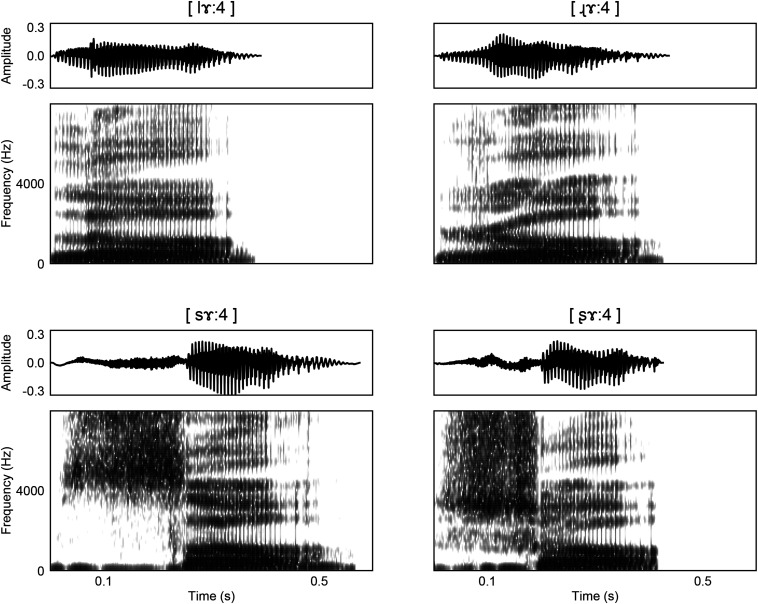
Waveforms and spectrograms for the (top) approximant pair [lɤː4]/[ɻɤː4] and the (bottom) fricative pair [sɤː4]/[ʂɤː4].

As demonstrated in Figure 1, the distinctive acoustic feature between retroflex/non-retroflex fricative is the range of spectral energy. Retroflection is associated with lower spectral energy ranges in both fricatives and affricates (Lee, 1999). In the approximant pair [l]/[ɻ], retroflection leads to a lower F3, which is similar to the differences between prevocalic /l/ and /r/ in American English (Polka and Strange, 1985). A lower F3 correlates with more “r”-color. The retroflex [ɻ] also has a larger difference between F4 and F5 than [l].

### EEG Acquisition

Subjects were seated in a sound-attenuated cabin. They were instructed to watch a silent movie while passively listening to the stimuli during the EEG recording (Tervaniemi et al., 1999; Scharinger et al., 2016b). Experimenters communicated with participants in Mandarin, and all experiment materials (i.e., instructions, recruitment and debriefing materials) were provided in Mandarin. Stimuli were presented in an auditory oddball paradigm. The experiment consisted of two blocks. One block contained retroflex standards and non-retroflex deviants. The other block had non-retroflex standards and retroflex deviants. The order of blocks was randomized for each subject. In each block, each of the four tokens of the deviant category was presented 20 times, totaling 80 deviant tokens per block. The order of deviant tokens was randomized. Prior to each deviant stimulus, a random number (drawn from a uniform distribution between 4 and 10) of standard tokens were presented. This resulted in an approximate standard-to-deviant ratio of 7-to-1. There were approximately 560 standard tokens per block. The standard tokens were also randomly sampled from the four stimuli of its category. The duration of the interstimulus interval was randomly sampled from a uniform distribution between 1.25 and 1.75 s. These values were selected to reinforce phonological-level processing (Werker and Logan, 1985; Yu Y. H. et al., 2017).

Continuous EEG signals were acquired with 32-channel ActiCAP active electrodes (Brain Products GmbH, Germany) and an actiCHamp (Brain Products GmbH, Germany) amplifier. The data were digitalized at 1000 Hz with a 0.01–500 Hz online bandpass filter. Electrodes were placed according to the international 10-20 system and positions include Fp1/2, F3/4, F7/8, FC1/2, FC5/6, FT9/10, C3/4, T7/8, CP1/2, CP5/6, TP9/10, P3/4, P7/8, O1/2, Oz, Fz, Cz, and Pz. A ground electrode was placed at Fpz. The continuous EEG signal was referenced to the left mastoid (TP9) online.

To ensure precise stimulus-digital trigger timing, auditory stimuli were first passed through a StimTrak device (Brain Products GmbH, Germany), which is engineered specifically for EEG trigger precision. In our configuration, the StimTrak device forward the auditory signal simultaneously to the amplifier and headphones. The auditory signal sent to the amplifier is recorded as an additional EEG channel. This provides the moment at which the auditory stimulus is presented to the participants. The auditory stimuli were delivered to subjects through BeyerDynamic DT 770 PRO headphones.

### EEG Analysis

Data analysis was conducted in MATLAB (Mathworks, Inc) using the EEGLAB toolbox (Delorme and Makeig, 2004). First, we corrected for any offset delays between the trigger and the auditory stimulus presentation to ensure millisecond-precise stimulus-digital trigger synchrony. This was done by cross-correlating the original stimuli sound files and the audio track in the EEG recording delivered by StimTrak. Subsequently, triggers were aligned with the onset of audio file in the EEG recording. Trigger-stimulus onset synchrony was checked in the raw continuous EEG signal. Next, the EEG signal was re-referenced to the linked mastoids, which provide the most robust MNN responses (Mahajan et al., 2017). The EEG signal was then filtered with a Hamming windowed sinc FIR filter. The signal was first high-pass filtered at 0.1 Hz (transition bandwidth 0.1 Hz), then low-pass filtered at 70 Hz (transition bandwidth 17.5 Hz). The filtered signal was downsampled to 250 Hz. The PREP pipeline (Bigdely-Shamlo et al., 2015) was subsequently used to remove line noise and bad channels. Then, the artifact subspace reconstruction (ASR^1^ algorithm was applied to remove stationary artifacts. On average, 2.3 channels were removed as bad channels. Next, previously removed channels were interpolated. An independent component analysis (ICA) decomposition was done by Adaptive Mixture Independent Component Analysis (AMICA; Palmer et al., 2012). Dipoles of the independent components were localized with the DIPFIT plug-in (Oostenveld and Oostendorp, 2002)^2^. We manually inspected the topography, fitted dipole locations, waveforms, and residual variances to identify the independent components. Independent components that correspond to eye movements (e.g., blinks, saccades) or widely distributed artifacts on the scalp were removed. Fewer than six (*x̄* = 2.1) independent components were removed for each subject. Next, the continuous EEG data were epoched with a 1-second pre-onset period and a 2-second post-onset period. Epochs with voltages ± 75 μV were rejected. Combined with the window rejection from the application of ASR, fewer than 3% of trials were removed for each subject. Among the standard trials, the two trials immediately after each deviant trial were excluded from further analysis. This was done to ensure that only trials where participants heard a sequence of standards prior to a deviant were included in the analysis. After preprocessing, each subject had more than 357 standards and more than 76 deviants in each of the two blocks for the final analysis.

Within-block MMN analyses compare the evoked potentials to the deviant and standard stimuli in the same experimental block. Different properties of the standards and deviants could elicit different ERPs even without the oddball frequency differences. This could potentially confound the MMN analysis. An alternative is to analyze the identity MMN (iMMN). In this analysis, the ERP to the deviant is compared with the ERP to the standard version of the same stimulus (Pulvermüller and Shtyrov, 2006; Peter et al., 2010; Hestvik and Durvasula, 2016). In the current study, we include both the within-block (see section “Within-Block MMN”) and iMMN (see section “Identity MMN”) analyses. Because MMNs are largest over fronto-central scalp areas when referenced to linked mastoids (Näätänen et al., 2007), the average potential of four fronto-central electrode sites (i.e., Cz, Fz, FC1/2) is used to calculate average ERPs. Statistical analyses are conducted in EEGLAB with permutation tests on the t-statistic and an FDR correction for multiple comparisons. Differences with pFDR < 0.05 are reported as statistically significant. In the ERP analysis, only significant differences longer than 30 ms in duration are reported and discussed. Because MMNs are sometimes accompanied by a polarity reversal at the mastoid sites (Schröger, 1998), we also computed and visually inspected the averaged difference waves at mastoid electrodes and fronto-central electrodes after average-referencing the data. In the topographic analysis, permutation tests for both the within-block and iMMN comparisons are conducted in each time window on each electrode site (with an FDR correction).

## Results

### Within-Block MMN

**FIGURE 2:**
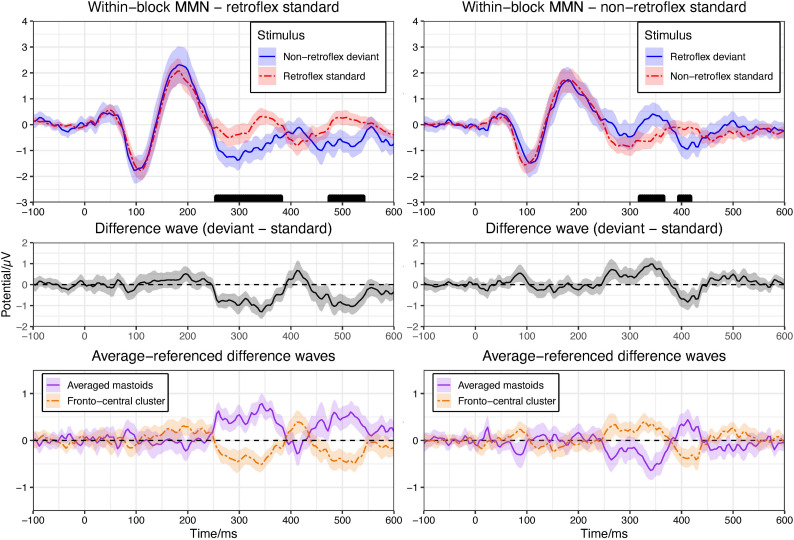
Upper panel: means of grand average ERPs at electrodes Cz, Fz, FC1/2. Ribbons represent 95% confidence intervals. Rug plots along the x-axes mark time points with significant differences for greater than 30 ms in duration (pFDR < 0.05). Plots are arranged to show the within-block MMNs. Left panel compares the ERPs to the retroflex standard and the non-retroflex deviant; right panel compares the ERPs to the non-retroflex standard and the retroflex deviant. Middle panel: difference waves of grand average ERPs at the fronto-central cluster (Cz, Fz, FC1/2). Lower panel: difference waves of grand average ERPs of averaged mastoids and the fronto-central cluster (Cz, Fz, FC1/2) with an average reference.

In the retroflex standard block, the permutation test in the −100–600 ms time window shows significant differences at 256–380 ms and 476–540 ms over fronto-central electrode sites. See Figure 2. The ERP to the deviant is more negative than to the standard, as is typically observed in MMN paradigms. In the non-retroflex standard block, the permutation test in the −100–600 ms time window shows significant differences between their grand average ERPs at 320–364 ms and 396–416 ms (Figure 2); however, the ERPs to retroflex deviants are more positive than the ERPs to non-retroflex standards at 320–364 ms and more negative at 396–416 ms. The positive difference to retroflex deviants at 320–364 ms is not consistent with the characteristics of an MMN.

To examine the nature of this positive difference to the retroflex deviant, we compared the ERPs in the block where the retroflex stimuli are the deviant to the ERPs in the block where the retroflex stimuli are the standard. The ERPs to the retroflex stimuli are similar across the two blocks, as are the ERPs to the non-retroflex stimuli. Thus, both the negative deflection to the deviant in the retroflex standard block and the positive deflection in the non-retroflex standard block may be at least partially caused by intrinsic differences in responses to the retroflex and non-retroflex sounds without the effect of presentation frequency. In other words, regardless of being the standard or the deviant, retroflex sounds elicited more positive ERPs around 320–364 ms, which may potentially confound the within-block MMN analysis. This observation motivated us to examine the iMMNs. Here, the ERP to a deviant is compared with the ERP to the same stimulus in the other block where it serves as the standard. This is done to ensure that potentially different ERPs to the two types of stimuli are controlled. Finally, we observed the typical mastoid reversal in auditory MMN studies; however, this was present in the retroflex standard block only and apparent between 252–388 ms, as well as 440–548 ms (see Figure 2).

### Identity MMN

**FIGURE 3:**
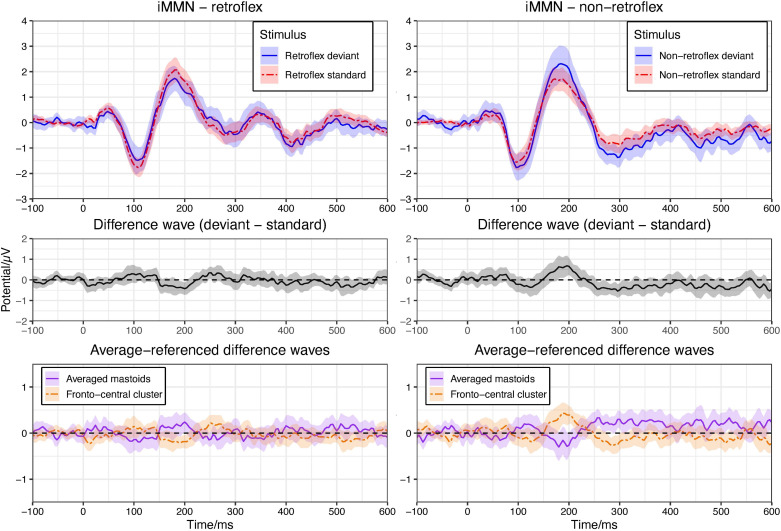
Upper panel: means of grand average ERPs at electrodes Cz, Fz, FC1/2. Ribbons represent 95% confidence intervals. In both panels, there is no significant difference between the ERPs at any time point (pFDR < 0.05). Plots are arranged to show iMMNs. Left panel compares the ERPs to the retroflex standard and deviant; right panel compares the ERPs to the non-retroflex standard and deviant. Middle panel: difference waves of grand average ERPs at the fronto-central cluster (Cz, Fz, FC1/2). Lower panel: difference waves of grand average ERPs of averaged mastoids and the fronto-central cluster (Cz, Fz, FC1/2) with an average reference.

Based on visual inspection, the average ERPs over front-central electrode sites to non-retroflex deviants are more negative than that to non-retroflex standards at most time points within the 250–550 ms time window. The ERPs to retroflex standards and deviants have a less defined relative positivity and do not differ throughout the time window; however, the permutation test in the −100–600 ms time window shows no significant differences in either the non-retroflex deviant/standard contrast or the retroflex deviant/standard contrast (Figure 3). The absence of significant negative deflections to non-retroflex deviants and positive deflections to retroflex deviants support the analysis that both deflections observed in the within-block MMN comparison are at least partially caused by the different ERPs to the retroflex and non-retroflex stimuli and not the standard-deviant relationship. Here, there is no such well-defined mastoid reversal as seen in the within-block MMN analysis. For non-retroflex stimuli, mastoid potentials trend positive, while frontal cluster potentials trend negative between 250–400 ms and 430–540 ms (see Figure 3).

### Topographic Comparison

**FIGURE 4:**
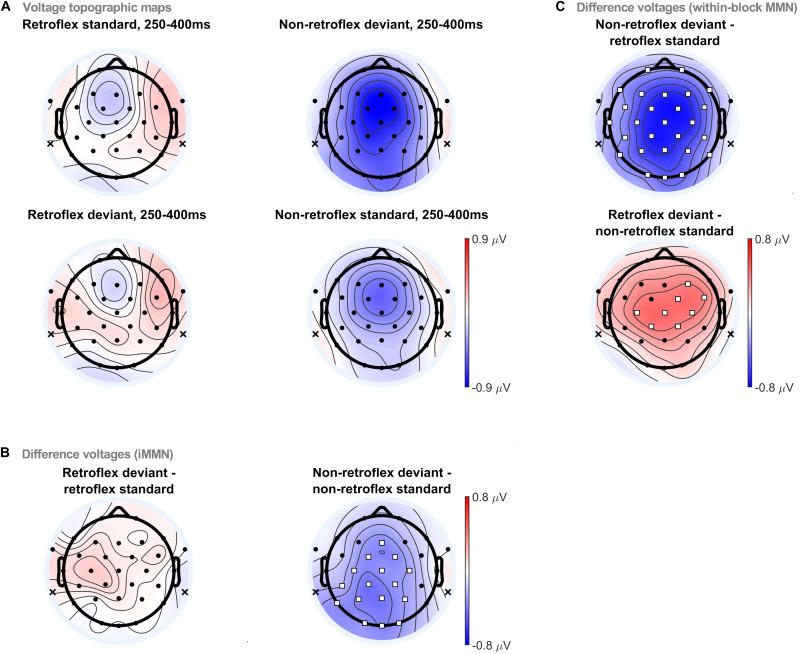
**(A)** Voltage topographic maps for retroflex/non-retroflex standard/deviant at 250–400 ms. Difference voltage topographic maps and the permutation test results for **(B)** within-block MMNs, **(C)** iMMNs. Electrodes highlighted in white squares denote electrode sites with significant differences using a permutation test (pFDR < 0.05). Topographic analyses were carried out with a linked mastoid reference. The reference locations are marked with an ‘x’ on the topographic plots.

Topographic comparisons were conducted in four time-windows: 150–250 ms, 250–400 ms, 400–450 ms, and 450–550 ms. The selection of these time-windows was largely based on visual inspection of the grand averaged evoked potentials. The 150–250 ms time-window is when MMNs typically occur. The 250–400 ms time-window is when we first observe differences in the within-block MMNs, as well as a negativity in non-retroflex iMMN. Moreover, it also includes the common time window for P300 (Pedroso et al., 2012). The 400–450 ms time-window is when changes in the within-block MMN polarities occur. Finally, the 450–550 ms time-window is the last time window with significant differences in within-block MMN comparisons. ERP topographies in the 250–400 ms time-window are provided in Figure 4. ERP topographies for other time windows are provided in the Supplementary Material. At 250–400 ms, permutation tests revealed significant differences elicited by non-retroflex deviants in both the within-block MMN and iMMN comparisons, as well as a significant difference elicited by retroflex deviants in the within-block MMN comparison. Non-retroflex deviants elicit more negative ERPs than both baseline conditions (i.e., retroflex standards and non-retroflex standards). This suggests that the negativities to non-retroflex deviants are not uniquely caused by differences between the ERPs to retroflex and non-retroflex stimuli, as the iMMN results suggest. Regarding the spatial distribution of the negativity, in the within-block MMN comparison, the negativity to non-retroflex deviants is distributed across all electrode sites except for FT9/10 and T8. In the iMMN non-retroflex comparison, the maximum negativity is distributed over left posterior sites and significant at fronto-central to occipital sites (i.e., Fz, FC1/2, Cz, C3/4, CP1/2, CP5, Pz, P3/4, P7, Oz, and O1/2). In Section 3.2, the iMMN comparison only considered fronto-central electrodes; as such, it would not have captured this more posterior distribution and could contribute to why we did not observe a significant difference in the non-retroflex comparison (see Figure 3). In other time-windows, the only significant difference in the iMMN comparisons is between 400–450 ms, where the non-retroflex deviant has a larger negative potential at Oz.

## Discussion

In the current experiment, Mandarin Chinese participants were presented with a varying standards paradigm that included inter-category variation. There were two experimental blocks. In the retroflex standard block, participants heard varying standards that differed in manner of articulation and shared only the feature [retroflex]. Deviants were the non-retroflex counterparts. In the non-retroflex standard block, the standards were the non-retroflex categories, and the deviants were the retroflex categories. No MMN studies, to our knowledge, have employed a varying standards paradigm wherein multiple phonetic categories are used as the standards. For an MMN to be elicited, the brain must extract the one common feature from the series of standards despite the significant inter-category variation. A binary feature account predicted equal-sized MMNs in both blocks, whereas a privative feature account predicted an asymmetric MMN. Assuming a privative account, we anticipated observing an MMN only in the retroflex standard block, where there is a common feature [retroflex] to be extracted. We also predicted no MMN in the non-retroflex standard block. This design allowed us to test whether listeners can identify and extract the common feature from a natural class set, which is a hallmark of feature behavior in phonetic and phonological systems.

In the within-block MMN analysis, we observed significant negative deflections in the retroflex standard block in the 256–380 ms and 476–540 ms time windows. In the non-retroflex standard block, we observed a more positive deviant response in the 320–364 ms time window. This polarity is opposite to the typical MMN response. In the iMMN analysis, we did not observe differences over fronto-central electrode sites between the responses to standards and deviants. We did, however, observe a difference in the non-retroflex standard-deviant comparison over central-parietal-occipital electrode sites in the 250–400 ms time window. These results are consistent with privative feature accounts and previous MMN studies of features (Eulitz and Lahiri, 2004; Scharinger et al., 2012; Cornell et al., 2013; Hestvik and Durvasula, 2016; Scharinger et al., 2016b; Schluter et al., 2016). In the following discussion, we first discuss issues related to stimulus selection in the many-to-many paradigm. Next, we discuss methodological considerations regarding the use of the within-block MMN and iMMN analyses and compare our results from these two analysis methods. Then, we discuss the delayed latency and the parietal scalp distribution of the negativity in the topographic analysis. Finally, we conclude with the broader implications for the feature [retroflex], specifically and phonological representations, more generally.

### Stimuli Variation

The current study utilizes a many-to-many oddball paradigm. The goal was to test Mandarin listeners’ ability to extract the [retroflex] feature from a series of standards that included inter-category variation. All retroflex consonants in Mandarin and their non-retroflex counterparts ([ʂ]/[s], [tʂ]/[ts], [tʂ^h^]/[ts^h^], [ɻ]/[l]) were included. This inter-category variation highlights a few issues worth discussing.

First, the average duration of retroflex stimuli is shorter and less variable than non-retroflex stimuli. In a pairwise comparison, the shorter duration of the retroflex stimuli is largely due to the pairs [ʂɤː4]/[sɤː4] and [tʂɤː4]/[tsɤː4]. This observation might reflect the state of affairs in natural speech or be due to chance in stimulus creation. The larger duration variation in the non-retroflex stimuli is principally due to [sɤː4] and [lɤː4], which have the longest and shortest durations, respectively, of all our items. These length differences could affect ERP latencies to our two stimulus types. In Figure 2, the N1 to retroflex stimuli is later than to non-retroflex stimuli in both blocks. Moreover, the larger duration variation in the non-retroflex stimuli could lead to a larger variation in the latency of peaks and troughs in ERPs and produce reduced amplitudes in the grand average ERPs. Both duration and its variation could influence the within-block MMNs. This is discussed in Section 4.2.

Second, our stimuli have different levels of frication. The approximants ([ɻ]/[l]) have a lower level of air turbulence than other stimuli (see Figure 1). It should be noted that this contrast in frication level is possible to elicit mismatch responses. The current study has a high ratio (1:3) of approximant to non-approximant stimuli and an uncontrolled number of intervening syllables between approximants in the experiment. This configuration renders a weak oddball ratio and reduces the likelihood of observing an MMN. These potential effects are left for future research. Another potential consideration is that [ɻ] is sometimes argued to be a fricative (Duanmu, 2007). Under this categorization, [l] would be the only approximant in our study and the approximant to non-approximant stimuli ratio would be a strong oddball ratio (1:7). There are few points to note, however. First, we do not observe clear frication in the [ɻ] in the current study (see Figure 1). This is consistent with previous observations that the Mandarin Chinese [ɻ] has little to no frication (Fu, 1956; Wang, 1979; Duanmu, 2007; Lee-Kim, 2014). Even if we were to assume that [ɻ] contains more frication than [l], the difference in frication between our two approximant stimuli (i.e., [l, ɻ]) would be far less than the difference between [ɻ] and the fricatives/affricates. Thus, it is unlikely that listeners will group [ɻ] with the fricative/affricate stimuli, as opposed to treating it as an approximant.

Finally, each of our syllables corresponds to different numbers of Mandarin words with varying lexical frequency. Overall, the retroflex stimuli correspond to a slightly higher number of words and have a much higher frequency. This is largely due to the unaspirated affricate pair ([tʂ]/[ts]), as [tʂɤː4] is the Mandarin demonstrative 这 ‘this’. Because this stimulus pair constitute one-fourth of the stimuli in the experiment and are randomly presented with other stimuli, the potential effect of occurrence frequency might be reduced. Lexical frequency has been shown to affect the MMN amplitude: High-frequency deviants elicit larger MMN amplitudes than low-frequency deviants (Alexandrov et al., 2011); however, the opposite pattern is observed in our experiment. The low-frequency (non-retroflex) stimuli elicit larger negativities. This likely suggests that the asymmetry in negativities in the current study is not due to lexical frequency.

### Within-Block MMN and iMMN

The within-block MMN design compares the ERPs to deviants and standards in the same block. The standard and deviant are drawn from different categories, for example, retroflex versus non-retroflex. If the ERPs to these different stimuli categories are intrinsically distinct and there is no effect of being a standard or deviant, then this could potentially confound the interpretation of the MMN. To control for this potential confound, some studies calculate an iMMN, which compares the standard and deviant versions of the same stimulus across experimental blocks (e.g., Kraus et al., 1995; Chandrasekaran et al., 2007; Peter et al., 2010; Hestvik and Durvasula, 2016). An alternative method to obtain the iMMN is to present the deviant stimulus alone in a separate control block and subtract the average ERP in this control block from the ERPs to the same deviant stimulus in an experimental block (Kraus et al., 1995; Sharma and Dorman, 2000; Pettigrew et al., 2004).

In our experiment, the feature [retroflex] is physically manifested differently across phonetic categories (e.g., lower distribution of spectral energy in fricatives and affricates, lower F3 and a larger difference between F4 and F5 in approximants, etc.). This results in varying amounts of acoustic differences between the retroflex stimuli and their non-retroflex counterparts. For instance, [ɻ] and [l] differ along more acoustic dimensions than the fricatives and affricates. The acoustic differences between standards and deviants in this experiment are shown to elicit different ERPs and produce unusual patterns (e.g., the unexpected positivity in the MMN time window) in the within-block MMN analysis. Therefore, we also conducted an iMMN analysis. With the assumption that [retroflex] is a privative feature, it is predicted that the retroflex iMMNs are absent or weaker than the non-retroflex iMMNs.

Although the iMMN calculation can eliminate the influence of intrinsic differences between responses to comparing stimuli of different categories, iMMNs are susceptible to the repetition effect of the same or similar stimuli. In the *adaption to standards* and *predictive coding* accounts of the MMN (see Fitzgerald and Todd, 2020 for a review), ERPs to standards might be modulated as a result of repetition. For example, the number of repetitions could influence ERPs to the standard in such a way that the “repetition positivity” (RP, a positive deflection in the ERP around 50 to 250 ms to the standard stimuli) increases with more repetitions (Haenschel, 2005). This increased RP leads to a larger MMN because the RP occurs at a similar window as the MMN, and the relatively more positive standard response results in a more negative difference wave. The RP, however, has only been consistently reported in the roving oddball paradigm. In a roving oddball paradigm, each block contains different trains of stimuli, and each train has a different standard and deviant. The deviant in the preceding train is the standard of the following train (Haenschel, 2005). In our experiment, because the roving oddball paradigm was not used, we do not *a priori* expect to observe the RP.

Besides the RP, another potential repetition effect is the refractory state of frequency-specific neurons (Jacobsen et al., 2003; Näätänen et al., 2005), which could reduce N1 amplitudes and contribute to the calculated MMNs. In the current study, retroflex stimuli tend to have higher energy in the 2000–4000 Hz frequency range, thus the neurons sensitive to this frequency range might generate a smaller N1 response when the retroflex stimuli are repeated as the standard. In turn, this could increase the amplitude of the calculated iMMN in the N1 time window. Assuming the refractory effect is present in our data, we can predict that retroflex standards elicit a smaller N1 relative to retroflex deviants. The result shows no significant positive deflections or any other significantly different responses to the retroflex standard relative to the retroflex deviant. Thus, the refractory effect—or any other repetition effect to the retroflex standard—is not observed in the ERPs to the retroflex standard. Note that although we focused on the RP wave and the refractory effect, the repetition of standards could also influence brain responses in other ways that should be considered when interpreting the iMMN, such as enhancing β-band oscillatory power (Scharinger et al., 2016b).

In summary, the positive difference to the retroflex deviant in the within-block MMN comparison and its reduction in amplitude/disappearance in the iMMN comparison confirmed that the brain responds to retroflex/non-retroflex stimuli differently and that it is insufficient to use the within-block MMN as the sole evidence for the negativity to the non-retroflex deviants; however, the survival of significant differences in the iMMN analysis for non-retroflex stimuli from multiple electrode sites in the topographic analysis suggests that non-retroflex deviants indeed elicit a negative response. The existence of negative deflections that only associate with the non-retroflex stimuli is also consistent with the observation that in a within-block MMN analysis (see Figure 2), the difference between the non-retroflex deviant and retroflex standard is larger than the difference between the retroflex deviant and the non-retroflex standard.

### Latency and Distribution of the Negativities

Negative deflections in this study occurred around 250–450 ms post-stimulus onset. The time window is later than the normal MMN window. Negativities in oddball paradigms distributed at a late time window have been reported as late MMNs (e.g., Korpilahti et al., 1995; Zachau et al., 2005) or late discriminative negativities (LDNs; Cheour et al., 2001; Čeponienė et al., 2002; Martynova et al., 2003; Strotseva-Feinschmidt et al., 2015; Hestvik and Durvasula, 2016). The LDN normally appears with the traditional MMN, but it can also appear independently (Strotseva-Feinschmidt et al., 2015). The LDN is more frequently found in children than in adults and is shown to decrease (Bishop et al., 2011) or disappear (Strotseva-Feinschmidt et al., 2015) with development into adulthood. In adults, the LDN spatial distribution is difficult to characterize. In the limited number of adult LDN reports, it has been observed in various locations, including anterior sites (Zachau et al., 2005; Hestvik and Durvasula, 2016), fronto-central sites (Korpilahti et al., 1995; Shafer et al., 2004), right and central sites (Peter et al., 2012), and parieto-central sites (Hestvik and Durvasula, 2016). Thus, discerning the presence of an LDN in adults based on topographic patterns is not straightforward.

The rarity of LDNs in adults leads to the question of what property of the stimuli elicits them. The cause of the LDN in adults is insufficiently studied and yet unknown. Bishop et al. (2011) suggest that LDNs might appear as a result of additional processing required by certain features of stimuli that are difficult to detect. Their hypothesis is based on the finding that in contrast to MMNs, LDNs are larger for smaller differences between standard and deviant stimuli (see also Čeponienė et al., 2004). This hypothesis can also account for the decrease of LDNs with age considering the maturation of brain and the exposure to language. For adults, LDNs can be elicited by both speech sounds (Hestvik and Durvasula, 2016; Monahan et al., in prep.) and non-speech sounds (Zachau et al., 2005; Peter et al., 2012). The experiments that elicited adult LDNs along with our experiment all use the many-to-many oddball paradigm (i.e., multiple unique stimuli for both standards and deviants). In our current paradigm, the variation in both standards and deviants demands a more abstract grouping for deviant detection. This would agree with the potential relationship between the presence of LDNs and processing difficulty. Thus, it is possible that a certain level of complexity in the stimuli is a necessary condition to elicit adult LDNs. Additionally, the [retroflex] feature might be more difficult to process due to its low frequency in languages. Shafer et al. (2004) conducted an MMN study with retroflex (i.e., /ɖa/) and bilabial stimuli (i.e., /ba/). Hindi speakers showed an MMN with a later peak latency when /ɖa/ was the standard (∼200–300 ms), compared to when /ba/ was the standard (∼100–250 ms). The later MMN latency when retroflex stimuli are the standard might be comparable to the long-latency negativities in the retroflex-standard block in our experiment. Moreover, their observed asymmetric MMN is also consistent with a monovalent [retroflex] feature. That being said, in Shafer et al. (2004), there exist two changes between the standard and deviant: a place change and manner of articulation change. This makes exclusively interpreting the role of [retroflex] difficult.

Besides the late timing, the negativity in the iMMN analysis also occurs at an unexpected distribution that spreads from central to occipital electrode sites with a maximum negativity at parietal sites, instead of the fronto-central region where the MMN is usually observed to have the largest amplitude. One possible explanation for the more parietal distribution is that the negativity to the deviants in this experiment is generated at different neural sources than conventional MMNs. According to the dual-generator model (Näätänen et al., 1978), MMNs originate from a principal source in primary auditory cortex that is responsible for the memory component in the deviant detection, and a secondary prefrontal source responsible for the additional attention directed to the deviation; however, because the standard stimuli vary across phonetic categories in our design, primary areas of auditory cortex alone might not be sufficient for the identification of the retroflex feature. Thus, the formation and violation of the memory trace for the retroflex feature might need to be completed at a later stage in speech processing, for example, at a location closer to the superior temporal sulci where phonological information is processed (Okada and Hickok, 2006; Hickok and Poeppel, 2007; Rauschecker and Scott, 2009; Vaden et al., 2010). Using fMRI, Scharinger et al. (2016a) observed that underspecified vowels (i.e., [e]) following specified vowels (i.e., [o]) in same-different word pairs resulted in stronger blood oxygenation level dependent (BOLD) responses in bilateral superior-temporal sulcus (STS). This is in comparison to when the first member of the same-different word pair included an underspecified vowel, and the second member included a specified vowel. These results place a locus of feature processing in STS. Moreover, as they note, these findings mimic the typical pattern observed in asymmetric MMN responses to specified and underspecified speech sounds: A larger MMN is observed to underspecified deviants following specified standards compared to the opposite orientation. In the current experiment, a larger MMN was observed when the standard was specified for [retroflex], as compared to when the standard was underspecified. Given the relatively sparse electrode array (32 channels) used in the current experiment, source analyses are not possible; however, previous combined EEG and hemodynamic experiments are potentially useful in linking the current results with STS activity.

Both the latency of the negativities and their more posterior distribution in the iMMN subtraction resemble those of the N400 response. The N400 response is typically described as a negative deflection to semantically incongruent stimuli at posterior electrode sites around 200–600 ms post-stimulus onset (Kutas and Federmeier, 2011). Unlike the low-level processing of auditory information in the MMN elicitation, the N400 elicitation requires access to higher-level semantic information, which is reflected in the N400’s different neural generators. Studies have shown that brain regions supposed to be related to semantic processing, such as the middle and superior temporal areas, the medial temporal lobe, and the prefrontal areas are involved in the N400 responses (Kutas and Federmeier, 2011). The similarities in the latency and distribution of the negativities in our experiment and the N400 raise the question of whether the many-to-many oddball paradigm elicits negativities in a similar manner as the elicitation of the N400. Recently, the N400 has been accounted for within predictive coding frameworks for language processing, akin to extant models of the MMN (Bornkessel-Schlesewsky and Schlesewsky, 2019). Moreover, while the choice of the longer ISI in our design was intended to reinforce phonological processing (Werker and Logan, 1985; Yu Y. H. et al., 2017), it is also possible that it permitted greater influence of lexical factors than initially intended (see Čeponienė et al., 1999, who argued that shorter ISIs in children lead to stronger auditory memory traces and consequently larger MMNs for putatively phonological contrasts). That being said, each of our items corresponds to approximately six different words and more than 12 senses, on average. As such, it is difficult to know which particular lexical item is activated by the participant on a given trial, making the observation of consistent N400 effects at the lexical level unlikely in our design. We leave this possibility for future research.

### Asymmetric MMNs and Underspecification

As with previous MMN studies that have identified a role for features in speech processing (Eulitz and Lahiri, 2004; Scharinger et al., 2012; Cornell et al., 2013; Hestvik and Durvasula, 2016; Scharinger et al., 2016b; Schluter et al., 2016; Hestvik et al., 2020), we observed an asymmetric MMN. In each of these previous studies, an MMN was observed when the category with a specified feature was the standard and either no MMN or a reduced MMN when the category underspecified for that feature was used as the standard. In the current paper, we did not test an underspecified relationship *per se*, but the presence or absence of a common feature, i.e., [retroflex], in a series of standards that varied in their phonetic category. In this sense, the current experiment is similar to these previous findings. For example, Cornell et al. (2013) tested [ɡ] versus [d]. As [coronal] is thought to be underspecified in the lexicon (Archangeli, 1988; Avery and Rice, 1989; Paradis and Prunet, 1991; Lahiri and Reetz, 2010; Cummings et al., 2017), [d] is underspecified for its place feature. The place feature [dorsal], however, is specified, and so [ɡ] has a specified place feature in the lexicon. Cornell et al. (2013) compared experimental blocks when [d] was the standard and [ɡ] was the deviant versus when [ɡ] was the standard and [d] was the deviant. A larger MMN was observed when [ɡ] is the standard, as [ɡ] is specified for its place feature. As such, when a privative feature is specified in the standards, and the deviant does not contain that feature, a mismatch occurs, resulting in an MMN. When the feature is underspecified, there is nothing upon which an auditory memory representation can be constructed that would contrast with the deviant. In these cases, there is no mismatch, and either no MMN or a smaller MMN is observed.

The current experiment is similar in that there is a single feature that can be extracted from the varying standards in the retroflex standard block, assuming that [retroflex] is a privative feature. And if the deviant mismatches with that feature, then an MMN and an iMMN is predicted, as we observed. When there is no shared feature, the deviant will not be able to mismatch with the auditory memory representation of the standard and again, either no MMN or a reduced MMN is predicted. We observed no MMN in both the within-block MMN and iMMN analyses in the non-retroflex standard block. It might be argued that predicting an asymmetric MMN is problematic because our non-retroflex standards were all [coronal] in their place of articulation. And as such, they shared a common feature, which should elicit an MMN to the retroflex deviants in the context of our coronal non-retroflex standards. Thus, the MMNs to the retroflex and non-retroflex stimuli should be symmetrical. There are two potential responses. First, each of our retroflex categories was also [coronal] in their place of articulation. Consequently, this is not a distinguishing feature between the retroflex and non-retroflex categories. That is, the feature [coronal] will not create a mismatch, which is necessary for an MMN (Lahiri and Reetz, 2010). Second, it has been demonstrated that when the standards are [coronal] and that is the distinguishing feature with the deviants, either no or reduced MMNs are observed, as [coronal] is underspecified in the mental lexicon (Eulitz and Lahiri, 2004; Cornell et al., 2013; Cummings et al., 2017). In light of this, symmetric MMNs based on [coronal] to retroflex and non-retroflex stimuli are unlikely. In short, we conclude that the feature [retroflex] is privative in Mandarin Chinese, and this feature can be extracted from a series of standards with inter-category variation that all share this feature.

## Conclusion

The goal of the current paper was to determine whether listeners can extract a common phonetic feature from a series of standards with inter-category variation in an MMN paradigm. Observing an MMN would suggest that listeners extracted the relevant phonetic feature, and more broadly, that listeners access and represent speech sounds in terms of features. No previous work, to the best of our knowledge, has employed inter-category variation in the standards. In particular, we presented Mandarin-speaking participants with two blocks in an auditory oddball paradigm. In one block, the standards all shared the retroflex feature and the deviants were non-retroflex consonants. In the other block, the standards were all non-retroflex. A binary model for distinctive features would predict symmetric MMNs across the two blocks. A privative model for distinctive features would predict an MMN only in the retroflex standard block and no MMN in the non-retroflex standard block, as there would be no feature to bind the standard stimuli together. We found late MMNs/LDNs in the retroflex standard block only. This result suggests that first, Mandarin speakers extract the privative feature [retroflex] from varying stimuli, and an asymmetric MMN was observed. This supports a privative model of distinctive features. Second, the later differences in the ERP responses in a many-to-many paradigm might suggest the extraction of a feature from inter-category stimuli requires additional effort. In summary, listeners can extract phonetic features and construct auditory memory traces based on these features—despite significant acoustic and inter-category phonological variation—so long as a shared feature binds the standards together.

## Data Availability Statement

The stimuli and raw EEG datasets can be found in the Open Science Framework https://osf.io/uyg9e/.

## Ethics Statement

The studies involving human participants were reviewed and approved by University of Toronto Research Ethics Board. The patients/participants provided their written informed consent to participate in this study.

## Author Contributions

ZF and PM designed the experiment, created thestimuli, analyzed the data and co-wrote the manuscript. ZF programmed the experiment and ran participants, supported by research assistants. Both authors contributed to the article and approved the submitted version.

## Conflict of Interest

The authors declare that the research was conducted in the absence of any commercial or financial relationships that could be construed as a potential conflict of interest.

## Footnotes

1 https://sccn.ucsd.edu/wiki/Artifact_Subspace_Reconstruction_(ASR)

2 https://sccn.ucsd.edu/wiki/A08:_DIPFIT
