# Supplementary material for: Extracting Phonetic Features From Natural Classes: A Mismatch Negativity Study of Mandarin Chinese Retroflex Consonants
Source: Front Hum Neurosci. 2021 Mar 24;15:609898. doi: 10.3389/fnhum.2021.609898 (PMC8029992; doi:10.3389/fnhum.2021.609898)
Supplement: Supplementary file 1 [file Data_Sheet_1.PDF]

Supplementary Material

Supplementary Table S1. pFDR-values for four series of permutation tests on means of grand average ERPs at electrodes Cz, Fz, FC1/2. The four series of permutation tests are two MMN comparisons (retroflex standard/nonretroflex deviant, non-retroflex standard/retroflex deviant) and two iMMN comparisons (retroflex standard/deviant, non-retroflex standard/deviant).

time (ms)	non-retroflex standard/deviant	retroflex standard/deviant	retroflex standard/non-retroflex deviant	non-retroflex standard/retroflex deviant
-100	1	1	1	1
-96	1	1	1	1
-92	1	1	1	1
-88	1	1	1	1
-84	1	1	1	1
-80	1	1	1	1
-76	1	1	1	1
-72	1	1	1	1
-68	1	1	1	1
-64	1	1	1	1
-60	1	1	1	1
-56	1	1	1	1
-52	1	1	1	1
-48	1	1	1	1
-44	1	1	1	1
-40	1	1	1	1
-36	1	1	1	1
-32	1	1	1	1
-28	1	1	1	1
-24	1	1	1	1
-20	1	1	1	1
-16	1	1	1	1
-12	1	1	1	1
-8	1	1	1	1
-4	1	1	1	1
0	1	1	1	1
4	1	1	1	1
8	1	1	1	1
12	1	1	1	1
16	1	1	1	1

20	1	1	1	1
24	1	1	1	1
28	1	1	1	1
32	1	1	1	1
36	1	1	1	1
40	1	1	1	1
44	1	1	1	1
48	1	1	1	1
52	1	1	1	1
56	1	1	1	1
60	1	1	1	1
64	1	1	1	1
68	1	1	1	1
72	1	1	1	1
76	1	1	1	1
80	1	1	1	0.09119268
84	1	1	1	0.09119268
88	1	1	1	0.07671581
92	1	1	1	0.09549486
96	1	1	1	1
100	1	1	1	1
104	1	1	1	1
108	1	1	1	1
112	1	1	1	1
116	1	1	1	1
120	1	1	1	1
124	1	1	1	1
128	1	1	1	1
132	1	1	1	1
136	1	1	1	1
140	1	1	1	1
144	1	1	1	1
148	1	1	1	1
152	1	1	1	0.09119268
156	1	1	1	1
160	1	1	1	1
164	1	1	1	1
168	1	1	1	1
172	1	1	1	1

176	1	1	1	1
180	1	1	1	1
184	1	1	1	1
188	1	1	1	1
192	1	1	0.07242022	1
196	1	1	0.02657201	1
200	1	1	0.01695232	1
204	1	1	1	1
208	1	1	1	1
212	1	1	1	1
216	1	1	1	1
220	1	1	1	1
224	1	1	1	1
228	1	1	1	1
232	1	1	1	1
236	1	1	1	1
240	1	1	1	1
244	1	1	1	1
248	1	1	1	1
252	1	1	1	1
256	1	1	0.00205737	1
260	1	1	0.00205737	0.09772147
264	1	1	0.00205737	0.06758181
268	1	1	0.00205737	0.02814812
272	1	1	0.00205737	0.00986266
276	1	1	0.00205737	0.0330765
280	1	1	0.00205737	0.09549486
284	1	1	0.00205737	1
288	1	1	0.00205737	1
292	1	1	0.00205737	1
296	1	1	0.00205737	1
300	1	1	0.00205737	1
304	1	1	0.00205737	1
308	1	1	0.00205737	1
312	1	1	0.00205737	1
316	1	1	0.00205737	0.0581788
320	1	1	0.00205737	0.02814812
324	1	1	0.00205737	0.01902301
328	1	1	0.00205737	0.01902301
332	1	1	0.00205737	0.01902301

336	1	1	0.00205737	0.00986266
340	1	1	0.00205737	0.00986266
344	1	1	0.00205737	0.00986266
348	1	1	0.00205737	0.00986266
352	1	1	0.00205737	0.00986266
356	1	1	0.00205737	0.00986266
360	1	1	0.00205737	0.00986266
364	1	1	0.00205737	0.02814812
368	1	1	0.00205737	1
372	1	1	0.00963793	1
376	1	1	0.03424006	1
380	1	1	0.03424006	1
384	1	1	0.06528521	1
388	1	1	1	1
392	1	1	1	1
396	1	1	1	0.03754694
400	1	1	1	0.02340827
404	1	1	1	0.03754694
408	1	1	0.06453715	0.02340827
412	1	1	0.02880444	0.00986266
416	1	1	0.02880444	0.01902301
420	1	1	0.05953533	0.09119268
424	1	1	1	0.09119268
428	1	1	1	0.0330765
432	1	1	1	0.0472797
436	1	1	1	1
440	1	1	1	1
444	1	1	1	1
448	1	1	1	1
452	1	1	0.08608648	1
456	1	1	0.07583678	1
460	1	1	0.07583678	1
464	1	1	0.09014776	1
468	1	1	0.09772147	1
472	1	1	0.06528521	1
476	1	1	0.00205737	1
480	1	1	0.00205737	0.02340827
484	1	1	0.00205737	0.02340827
488	1	1	0.00205737	1

492	1	1	0.00205737	1
496	1	1	0.00205737	0.02340827
500	1	1	0.00205737	0.01902301
504	1	1	0.00205737	0.0472797
508	1	1	0.00205737	1
512	1	1	0.00205737	1
516	1	1	0.00205737	1
520	1	1	0.00205737	1
524	1	1	0.00205737	1
528	1	1	0.00205737	1
532	1	1	0.00600868	1
536	1	1	0.00963793	1
540	1	1	0.02038493	1
544	1	1	1	1
548	1	1	1	1
552	1	1	1	1
556	1	1	1	1
560	1	1	1	1
564	1	1	1	1
568	1	1	1	1
572	1	1	1	1
576	1	1	0.09119268	1
580	1	1	1	1
584	1	1	1	1
588	1	1	1	1
592	1	1	0.06758181	1
596	1	1	0.02340827	1
600	1	1	1	1

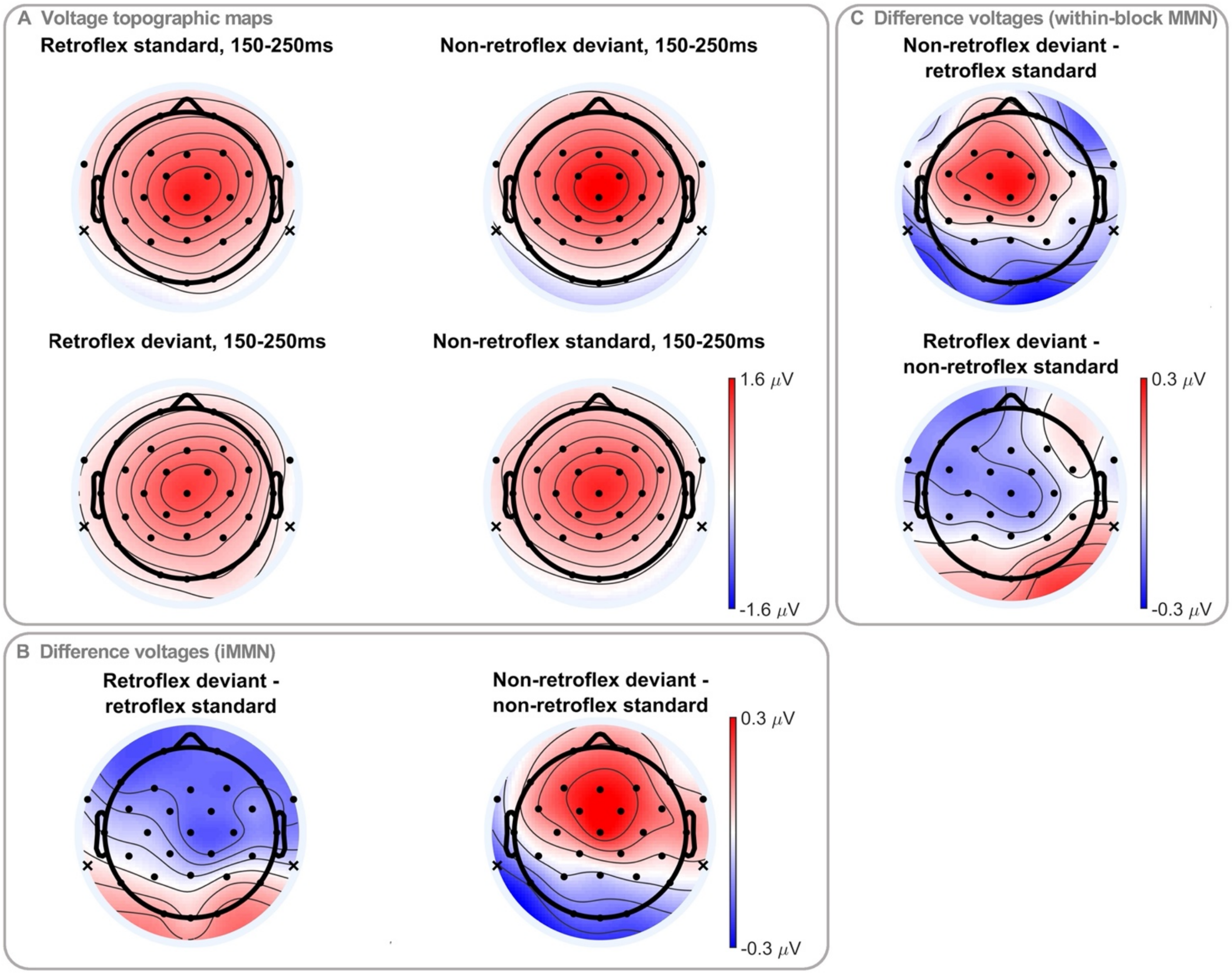

Supplementary Figure S2. (**A**) Voltage topographic maps for retroflex/non-retroflex standard/deviant at 150–250 ms. Difference voltage topographic maps and the permutation test results for (**B**) within-block MMNs, (**C**) iMMNs. Electrodes highlighted in white squares denote electrode sites with significant differences using a permutation test (pFDR < 0.05). Topographic analyses were carried out with a linked mastoid reference. The reference locations are marked with an ‘x’ on the topographic plots.

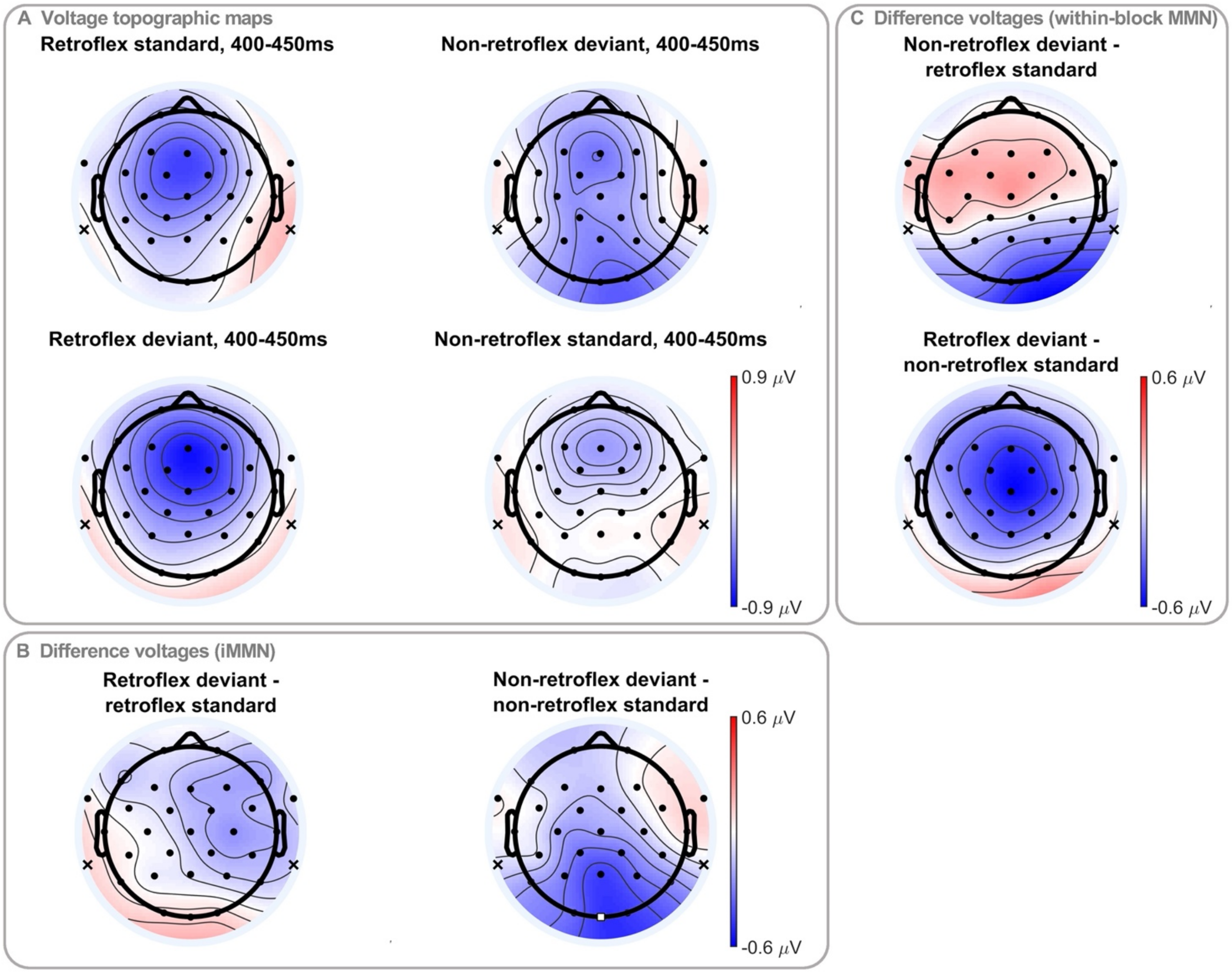

Supplementary Figure S3. (**A**) Voltage topographic maps for retroflex/non-retroflex standard/deviant at 400–450 ms. Difference voltage topographic maps and the permutation test results for (**B**) within-block MMNs, (**C**) iMMNs. Electrodes highlighted in white squares denote electrode sites with significant differences using a permutation test (pFDR < 0.05). Topographic analyses were carried out with a linked mastoid reference. The reference locations are marked with an 'x' on the topographic plots.

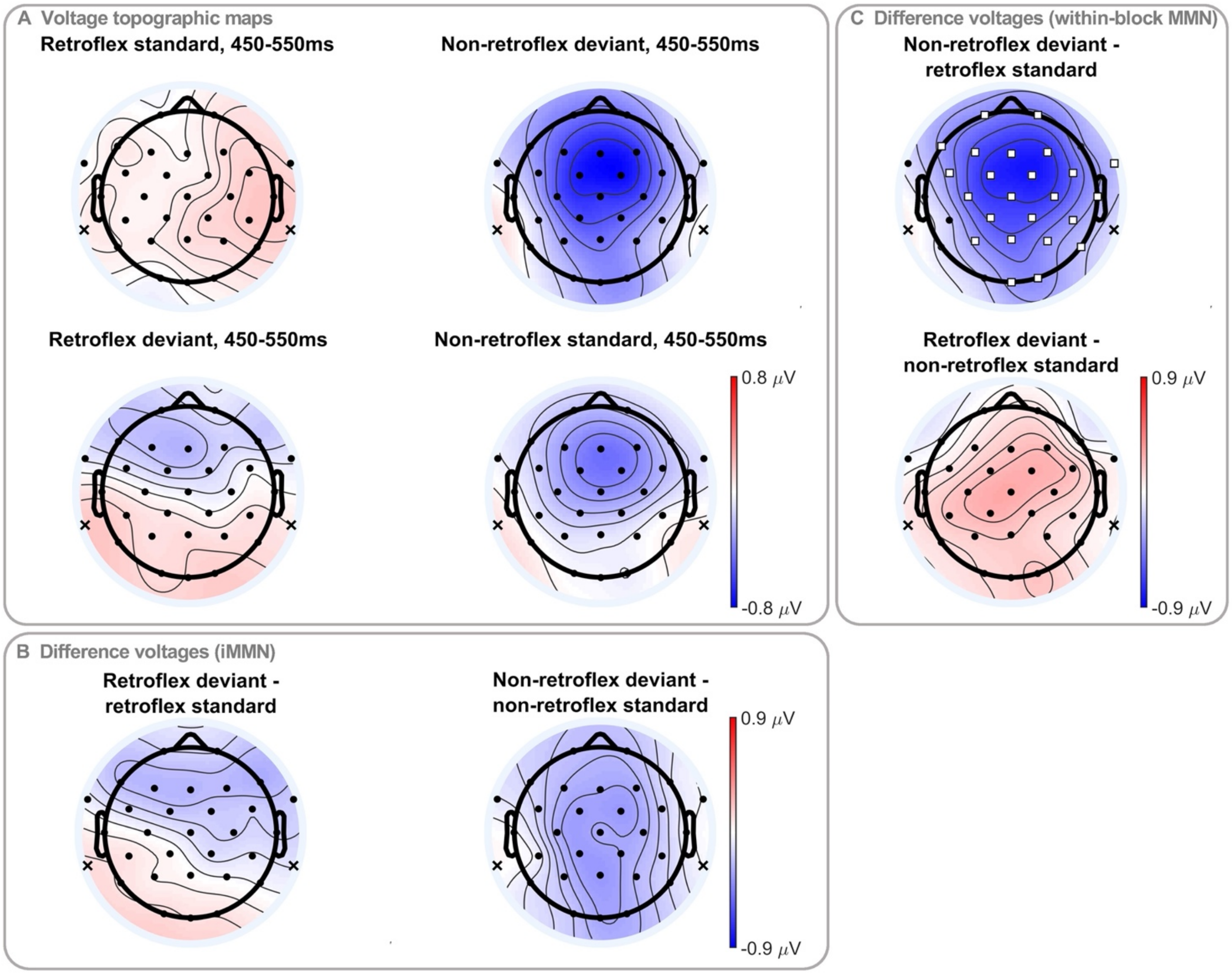

Supplementary Figure S4. (**A**) Voltage topographic maps for retroflex/non-retroflex standard/deviant at 450–550 ms. Difference voltage topographic maps and the permutation test results for (**B**) within-block MMNs, (**C**) iMMNs. Electrodes highlighted in white squares denote electrode sites with significant differences using a permutation test (pFDR < 0.05). Topographic analyses were carried out with a linked mastoid reference. The reference locations are marked with an 'x' on the topographic plots.
